# A randomized proof-of-mechanism trial of TNF antagonism for motivational anhedonia and related corticostriatal circuitry in depressed patients with high inflammation

**DOI:** 10.21203/rs.3.rs-3957252/v1

**Published:** 2024-03-05

**Authors:** Michael Treadway, Sarah Etuk, Jessica Cooper, Shabnam Hossein, Emma Hahn, Samantha Betters, Shiyin Liu, Amanda Arulpragasam, Brittany DeVries, Nadia Irfan, Makiah Nuutinen, Evanthia Wommack, Bobbi Woolwine, Mandakh Bekhbat, Philip Kragel, Jennifer Felger, Ebrahim Haroon, Andrew Miller

**Affiliations:** Emory University; Emory University; Emory University; Emory University; Emory University; Emory University; Emory University; Emory University; Emory University; Emory University; Emory University; Emory University; Emory University; Emory University; Emory University; Emory University; Emory University; Emory University School of Medicine

**Keywords:** Motivational Anhedonia, Inflammation, Cytokines, fMRI

## Abstract

Chronic, low-grade inflammation has been associated with motivational deficits in patients with major depression (MD). In turn, impaired motivation has been linked to poor quality of life across psychiatric disorders. We thus determined effects of the anti-inflammatory drug infliximab–a potent tumor necrosis factor (TNF) antagonist–on behavioral and neural measures of motivation in 42 medically stable, unmedicated MD patients with a C-reactive protein > 3mg/L. All patients underwent a double-blind, placebo-controlled, single-dose, randomized clinical trial with infliximab (5mg/kg) versus placebo. Behavioral performance on an effort-based decision-making task, self-report questionnaires, and neural responses during event-related functional magnetic resonance imaging were assessed at baseline and 2 weeks following infusion. We found that relative to placebo, patients receiving infliximab were more willing to expend effort for rewards. Moreover, increase in effortful choices was associated with reduced TNF signaling as indexed by decreased soluble TNF receptor type 2 (sTNFR2). Changes in effort-based decision-making and sTNFR2 were also associated with changes in task-related activity in a network of brain areas, including dmPFC, ventral striatum, and putamen, as well as the functional connectivity between these regions. Changes in sTNFR2 also mediated the relationships between drug condition and behavioral and neuroimaging measures. Finally, changes in self-reported anhedonia symptoms and effort-discounting behavior were associated with greater responses of an independently validated whole-brain predictive model (aka “neural signature”) sensitive to monetary rewards. Taken together, these data support the use of anti-inflammatory treatment to improve effort-based decision-making and associated brain circuitry in depressed patients with high inflammation.

## INTRODUCTION

Deficits in reward motivation–often referred to as “motivational anhedonia”^1–3^–are a common feature of mood and other psychiatric disorders and are strongly associated with impaired quality of life^4^. Prior human and laboratory animal studies have repeatedly linked motivational anhedonia to dysfunction of corticostriatal reward networks^5–7^ that support effortful behavior^8–10^ and include dopamine (DA)-rich areas such as the striatum as well as brain regions encompassing the dorsomedial prefrontal cortex including the dorsal anterior cingulate cortex and surrounding paracingulate and pre-supplementary motor areas (herein referred to collectively as “dmPFC”). Human functional imaging studies as well as animal lesion studies have repeatedly implicated the dmPFC as a critical hub for effort-based decision-making ^11–15^, which appears to encode multiple decision-variables related to effort cost^16^, choice difficulty^17^ and effort-related expectation violation^11, 18^. Taken together, the dmPFC and striatum have been found to encode distinct decision-variables related to effort-based choice and appear to be causally involved in the willingness to expend effort for rewards.

A substantial body of evidence suggests that inflammation may have a significant impact on brain regions involved in effort-based motivation in patients with major depression (MD). Elevations in peripheral inflammatory cytokines are often present in MD patients^19–22^ and have been reliably associated with symptoms of anhedonia ^23^. Moreover, inflammatory stimuli have been shown to exert direct effects on dmPFC activity and striatal DA availability in laboratory animals and humans. In the case of dmPFC, prior functional magnetic resonance imaging (fMRI) and magnetic resonance spectroscopy studies have found that administration of interferon (IFN)-alpha, typhoid vaccination, or endotoxin leads to decreased task-based activation^24–26^ as well as increased glutamate^27^. Similarly for striatal DA, studies in rodents, non-human primates and humans have found that the cytokines interleukin (IL)-1 beta, IL-6, and IFN-alpha lead to substantial decreases in DA^28–31^. Functional neuroimaging studies have also revealed attenuated activity in striatum during DA-sensitive processing, such as during reward anticipation or reward prediction errors following immune activation^32–35^.

These data suggest that effects of inflammatory signaling on corticostriatal circuitry may represent a specific pathophysiological substrate for motivational deficits in MD. Importantly, only ~ 30% of MD patients exhibit increased inflammation, possibly representing an “inflammatory subtype” for motivational deficits^21^. Consistent with this notion, evidence suggests that targeted DAergic or anti-inflammatory therapies exhibit selective benefit for symptoms of anhedonia in MD patients exhibiting high–but not low–inflammation^36, 37^. To date, however, no study has tested the hypothesis that inhibition of inflammation can reverse objective measures of motivational anhedonia and related mesolimbic circuitry in depressed patients with high inflammation.

## METHODS

### Participants

131 were assessed for eligibility, and 42 experiencing a current MD episode as determined by Structured Clinical Interview for DSM-5^38^ and a CRP > 3mg/L were randomized (see Figure S1 for consort diagram). Randomized participants were free from all psychotropic and anti-inflammatory medications for at least 4 weeks and were without evidence of chronic infection, autoimmune or inflammatory disorders, or unstable medical illnesses as determined by medical history, physical exam and laboratory testing (full details of eligibility criteria and assessment are included in the Supplemental Materials). No patient was removed from psychotropic treatment for the purposes of the study. Of the randomized participants, 38 had available self-report measures, inflammatory markers, and behavioral data from the EBDM task, and 37 had available neuroimaging data. Two additional participants exhibited significant motion (3mm-6mm) during task-based fMRI; one additional subject exhibited a low calibration response at 14 days. These subjects were included in analyses, but sensitivity analyses were performed to assess their impact on reported results (see Supplemental Materials). Finally, two participants had an insufficient number of trials for division into training and test datasets, and were therefore excluded from the multivariate analysis.

Demographic and clinical data of randomized participants are presented in Table 1 and a full consort diagram is provided in **Supplemental Figure S1**. Written informed consent was obtained from all participants. The Emory Institutional Review Board granted study approval (IRB00087941). There were no serious adverse events associated with this study. A full list of adverse events is included in the Supplemental Materials.

### Study Design

**Figure 1:**
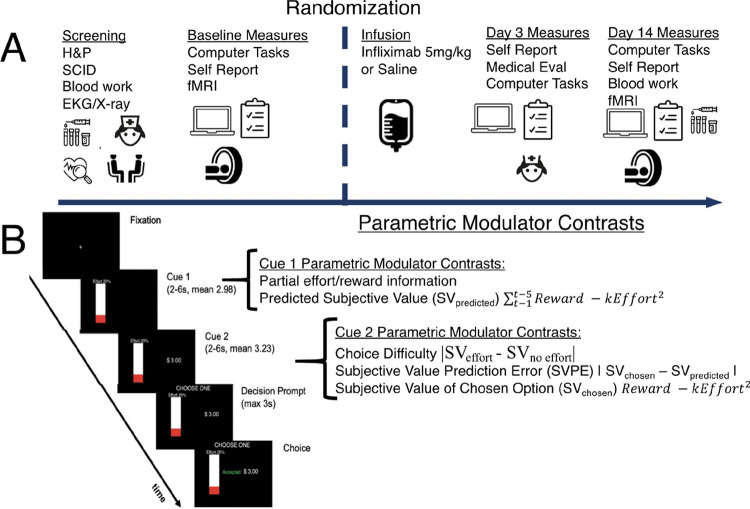
**A.** Schematic diagram of study design. All participants were screened for eligibility criteria, and eligible participants completed baseline symptom assessments and laboratory blood work. A baseline fMRI scanning session was followed by randomization and assessments at three days and at 14 days, when participants returned for a second fMRI session. Optional 24-hour and 7-day symptom and blood sample assessments were also collected (not shown). **B.** Schematic diagram of our previously developed EBDM task^11, 39^ with descriptions of parametric modulator contrasts used at either the Cue 1 or Cue 2 epoch of each trial (see Online Methods for details). *Abbreviations*. H&P: history and physical; SCID: structured clinical diagnostic interview; SV: subjective value; SVPE: subjective value prediction error.

The study utilized a randomized, placebo-controlled, clinical trial design to examine the effects of a single-dose of the TNF antagonist infliximab on behavioral and brain measures of motivational anhedonia. Baseline blood, behavioral, self-report and neuroimaging assessments were followed by an infusion of either a 5mg/kg of infliximab or saline (placebo), administered over ~ 40 minutes from saline bags matched for color and consistency. Blood samples and behavioral and self-report measures were repeated at 3 and 14 days, and neuroimaging assessments including resting-state fMRI and an effort-based decision-making task^11^ were repeated at 14 days (see Fig. 1A). All study personnel were blinded to group assignment. The randomization and blind key were tracked by the Emory Investigational Drug Service.

### Effort Based Decision-Making Task (EBDM)

The EBDM task is an fMRI-adapted effort-based decision-making task ^11, 39^ that measures neural responses to effort and reward magnitude. During each trial, participants were given the choice between High Effort and No Effort options. The High Effort option requires more effort (as measured by button presses) than the No Effort option. The reward obtained from the No Effort option was always $1 while rewards from the High Effort option varied between $1.00 and $5.75. The magnitude of effort required in the High Effort option consisted of 20%, 50%, 80%, and 100% of the participant’s maximal effort. The task shows good internal consistency, with a split-half reliability r = .94. Participants made choices in the scanner with the effort completed as soon as the scanning session was concluded. As such, the task can be viewed as measuring the choice to commit to effort expenditure for reward in the near future (see Supplemental Materials).

Clinical Assessments: Measures of motivational anhedonia included the reduced motivation (RM) subscale of the Multidimensional Fatigue Inventory (MFI^40^), a composite of items from the Motivation and Pleasure Scale-Self Report focused on effort^41^, and the anhedonia subscale from the Inventory of Depressive Symptomatology-Self Reported (IDS-SR^42^). Scales were collected prior to infusion at either screening, baseline MRI or infusion visits, and then at three days and 14 days post-infusion. These scales were pre-registered (https://osf.io/r6m49/) as clinical measures of motivational anhedonia given prior associations in inflammation and striatal function^31, 43^.

### Biological Assays

Whole blood was collected into EDTA-containing vacutainer tubes through indwelling catheters after 30 minutes of rest to limit effects of stress. Plasma was isolated and stored at −80 until batched assay. Customized Fluorokine MAP Multiplex Human Biomarker Panels (R&D Systems, Minneapolis, MN) were used to measure plasma soluble tumor necrosis factor receptor 2 (sTNFR2) and other inflammatory markers (see Supplemental Materials). Inter- and intra-assay coefficients of variation were reliably less than 10%, and no values were below limits of detection. Plasma CRP was measured using a high sensitivity turbidimetric assay in the CLIA-certified Emory Medical Laboratory.

Neuroimaging Data Acquistion: Functional and structural neuroimaging data were acquired on a Siemens 3T Tim Trio using a 32-channel phased-array head coil. Trial presentations were synchronized to initial volume acquisition. Functional (T2* weighted) images were aquired using a multiband sequence with the following sequence parameters: 3-mm^3^ isotropic voxels, repetition time (TR) = 1.0 s, echo time (TE) = 30 ms, flip angle (FA) = 65°, 52 interleaved axial slices, with slice orientation tilted 18° relative to the AC/PC plane to improve the temporal signal-to-noise ratio (tSNR) and minimize signal dropout of ventromedial prefrontal cortex. At the start of the imaging session, a high-resolution structural volume was also collected, with the following sequence parameters: 2-mm × 1-mm × 1-mm voxels, TR = 1.9 s, TE = 2.27 ms, FA = 9°.

### Power analysis and Impact of the Covid-19 Pandemic

Data in this study were drawn from a clinical trial (NCT03006393) focused on examining the effects of a single-dose of infliximab on brain function. The study was actively recruiting at the outset of the COVID-19 pandemic, at which point the investigators deemed continuation of the study to be unsafe, given the immune-suppressive effects of infliximab. Consequently, we were unable to achieve our originally proposed recruitment target of n = 80. As a result, the current study retains adequate power to detect large effect-sizes, but does not have adequate power for small or medium effect sizes, which may have increased type II error for some analyses. We note that prior pharmacologic studies of effort-based decision-making tasks have suggested large effect sizes (d > 1.0)^44, 45^, though these studies did not use infliximab.

Pre-registration: A pre-registered analysis plan that details hypotheses, key dependent variables, and primary methods can be found at https://osf.io/r6m49/. The current paper focuses on a subset of these data. Specifically, given the focus of the current paper on effects of infliximab on motivational circuitry, we used the secondary behavioral endpoint due to its temporal proximity to change in neuroimaging. Analyses not pre-registered are designated as “exploratory”. Table S1 in the Supplemental Materials summarizes our primary, secondary and exploratory analyses.

### General Statistical Methods

Multiple linear regression was used to examine associations between change from baseline to endpoint across choice data, clinical measures, sTNFR2 and fMRI data extracted from regions-of-interest (ROIs). To assess the effects of infliximab on inflammatory markers, multivariate analysis of variance (MANOVA) was used. Of note, sTNFR2 was the inflammatory cytokine that exhibited the greatest change following infliximab versus placebo and was thus used as the primary immune endpoint as well as a proxy for TNF signaling in the statistical analyses (see Supplemental Materials). For longitudinal analyses, difference score distributions of sTNFR2 appeared parametric, and raw values were used for computation of difference scores unless otherwise noted. To assess change in anhedonia symptoms or effort discounting (*k*), an ANCOVA was used with drug condition as a fixed factor and baseline values along with demographic variables as covariates. For choice behavior on the EBDM task, a repeated measures ANOVAs was used in lieu of an ANCOVA to model the interactions between time, drug condition and individual effort levels. Greenhouse-Geisser corrections are reported in cases where the sphericity assumption was violated. All statistical analyses included sex and age as covariates unless otherwise specified. All statistical analyses are two-tailed unless pre-registered as a one-tailed test or otherwise noted.

### Neuroimaging Preprocessing and Data Quality Evaluation

For all neuroimaging preprocessing and first-level GLM analysis, we used SPM12 (Wellcome Department of Imaging Neuroscience, Institute of Neurology, London, UK). SPM12 preprocessing included realignment estimation and implementation, co-registration to the individual’s high resolution structural scan, normalization to MNI space, and spatial smoothing using a Gaussian kernel (6mm FWHM). To control for motion and other artifacts, data were visually inspected, and 6 realignment parameters were included as covariates for all GLM analysis. Visual inspection revealed 2 participants showing evidence of significant motion (> 3mm) that were subsequently examined as potential high-influence data points. Additionally, the GLM contrasts used in univariate and multivariate analyses were evaluated for multivariate outliers using the Mahalanobis distance. The impact of subjects or sessions flagged as potential outliers using this criterion were evaluated in a series of sensitivity analyses that included participants with poor motion and/or multivariate outliers as covariates and they were not found to alter the significance of any reported neuroimaging associations (see Supplemental Materials for Sensitivity Analyses).

Neuroimaging Analysis – First Level General Linear Models (GLM): For all first-level GLMs, the canonical HRF was used, and event durations were modeled based on the duration of each cue for each trial with SPM default orthogonalization. Based on our pre-registered analyses and our prior work^11, 39^, we examined change (14 day vs. baseline scans) across 7 parametric modulator contrasts: effort level at Cue 1, reward magnitude at Cue 1, predicted subjective value at Cue 1 (SV_predicted_), choice difficulty at Cue 2, subjective value of the chosen option (SV_chosen_) at Cue 2, a subjective value prediction error (SVPE) and choice “shifts” at Cue 2 (Fig. 1B).

The parametric modulator contrasts for Cue 1 were defined as follows:

- *Contrast 1: Effort magnitude*. The amount of effort required for a given trial (20%, 50%, etc.).
- *Contrast 2: Reward magnitude*. The amount of reward offered for a given trial ($1.00~$5.00).
- *Contrast 3: SV*_*predicted*_. As in our prior work^11, 39^, SV_predicted_ was defined using a sliding window analysis of previously-experienced subjective values of the same trial type. Therefore, the SV_*predicted*_ for a trial that began by presenting 20% effort at Cue 1 would be calculated as the running average of the most recent SV values for trials that included 20% effort.

The parametric modulator contrasts for Cue 2 were defined as follows:

- *Contrast 4: SV*_*chosen*_. The SV_chosen_ was calculated as the subjective value of the option
- *Contrast 5: SVPE*. The subjective value prediction error (SVPE) regressor was estimated by calculating the absolute value of the difference between the SV_chosen_, and the SV_predicted_.
- *Contrast 6: Choice Difficulty*. A parametric modulator calculated as the difference between the subjective value of the effortful and non-effortful options. Choices for which this difference was small indicated greater choice difficulty (because of similar values for both options).
- *Contrast 7: Choice Shift*. A parametric modulator coding a “1” if the choice on the prior trial was the same as the choice made on the current trial, and “0” if not.

A full description of results from all *a priori contrasts* is included in the Supplemental Materials.

In addition to the parametric modulators described above, we estimated single-trial models for the purpose of multivariate, beta-series and brain signature analyses. For these analyses, a trial specific beta-weight was estimated for the Cue1 onset, the Cue2 onset and the decision-phase onset of each trial. Analyses using single trial models focused on the Cue1 timepoint.

### Neuroimaging Analysis – Second Level Univariate GLMs

For comparison of Day 14 and baseline scans, the two task runs from each timepoint were concatenated into a single first-level GLM. A second-level contrast of [−1 −1 1 1] was then used to compare parametric modulators during the two baseline timepoint runs to the two 14 day timepoint runs. To examine the effects of infliximab, change in acceptance of 100% effort trials, change in effort discounting (*k*) and change in sTNFR2 levels, each of these variables were separately entered into second-level GLM that also included sex and age as covariates. For whole-brain analysis, correction for multiple comparisons was obtained using cluster-correction as implemented in SPM12, with an uncorrected height threshold of p < 0.001. For ROI mean betaweights from all voxels in each ROI was averaged and analyzed.

Neuroimaging Analysis – Univariate ROI Analysis: Our analysis plan identified five *a priori* regions of interest: the dmPFC, bilateral insula, nucleus accumbens (NAcc) and ventromedial prefrontal cortex (vmPFC). Masks for these ROIs were drawn from a prior functional parcellation of medial prefrontal cortex^46^, the Glasser atlas^47^ and the Harvard-Oxford atlas^48^. For ROI analyses, the first eigenvariate of mean betaweights from all voxels within each mask was extracted for each participant and used in subsequent analysis.

### Neuroimaging Analysis – Multivariate ROI Analysis

As an exploratory analysis, change in striatal sub-regions for the SV_predicted_ and SV_chosen_ was used to classify drug condition assignment. A cross-validated 3-fold classification with partial least squares regression was employed to estimate the area under the receiver operating characteristic (AUROC) for drug classification based on activity in bilateral ventral striatum, putamen and caudate as defined by the Pauli basal-ganglia atlas^49^. Only neuroimaging data was used (i.e., age and sex covariates were not included). The k-fold procedure was estimated 10 times for each region, with the mean AUROC across each fold and iteration used to estimate predictive performance for each region. For statistical inference, we estimated the mean squared error (mse) of classification for each ROI and compared it to a null distribution of 5,000 mse values generated by a randomly permuted neuroimaging data.

### Neuroimaging Analysis – Beta Series Correlation (BSC)

To understand how infliximab-induced changes in dmPFC responses during EBDM may drive network-level changes within corticostriatal circuits, we examined task-based functional connectivity (beta-series correlation; BSC). To assess changes in BSC, we first isolated the time series of beta-weights at Cue 1 for each ROI for each participant. We focused on Cue 1 for this analysis as it would detect changes prior to decision-outcome, and thereby could reveal network-level changes related to processing of partial information that would not be confounded by differences in the proportion of effortful options accepted between the two drug conditions. We then estimated the Pearson correlation between dmPFC and target striatal regions (VS, putamen, caudate). Resulting correlations were Fisher transformed to create a difference score in the BSC between each pair of nodes (ΔBSC). These ΔBSC scores were used as dependent variables in Ordinary Least Squares (OLS) regression analyses and bootstrapped-mediation analyses as described above with drug condition or ΔsTNFR2 used as predictor variables.

Neuroimaging Analysis – Reward Signature Comparison The goal of this analysis was to evaluate the similarity between change in neural activity during EBDM (14 day – baseline) and a pre-trained ‘reward signature’ developed to classify monetary wins during losses during gambling and monetary incentive delay tasks^50^. We first calculated the change in cosine similarity between each participant’s 14day-baseline mean contrast image and a neural signature. Regression was used to assess the relationship between the resulting cosine similarity and drug condition, ΔsTNFR2, Δ*k*, and Δ100% Effort as well as self-report measures of anhedonia.

## RESULTS

### Effects of infliximab on behavioral and clinical measures of motivational anhedonia.

**Figure 2:**
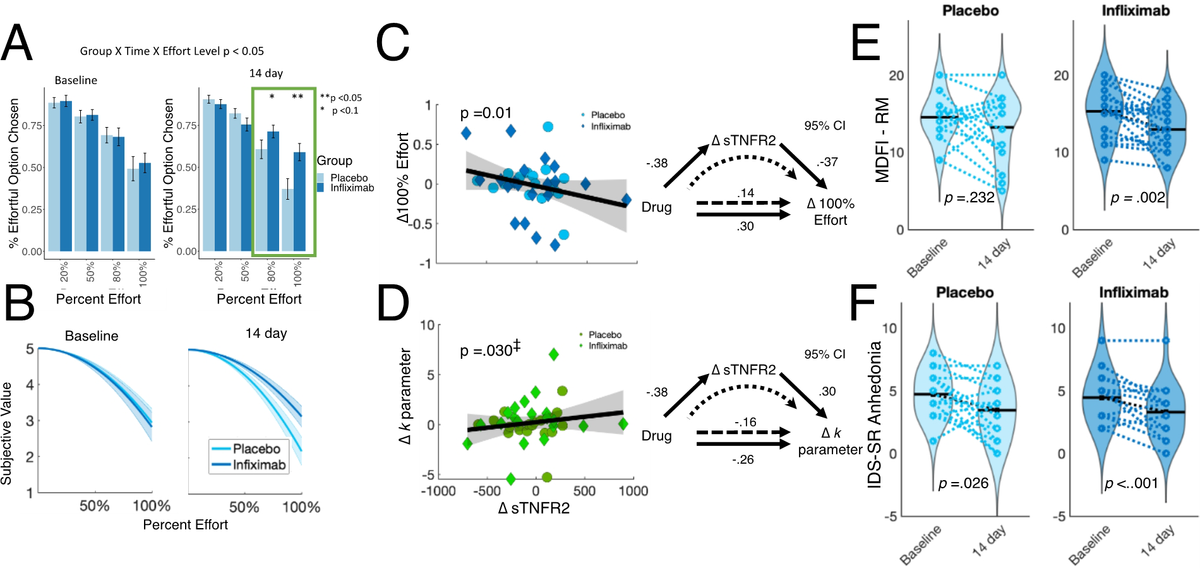
Effects of infliximab on effort-based decision-making. **A.** Group × Time × Effort level interaction in EBDM task performance. Relative to the placebo group, patients receiving infliximab accepted more effortful options at the two highest effort levels at 14 days. **B.** Group × Time interaction on effort discounting. Relative to the placebo group, patients receiving infliximab showed a reduction in effort discounting (indicated by the parameter *k*) at 14 days. **C.** A reduction in sTNFR2 at 14 days was associated with increased willingness to accept effortful options at the highest effort level (100%). **D.** A reduction in sTNFR2 at 14 days was associated with a reduction in effort discounting (*k*). These associations mediated the relationship between drug condition and effort performance. **E.** Changes in the MDFI Reduced Motivation Subscale for each group, with lower scores indicating greater improvement. **F.** Changes in the IDS-SR anhedonia subscale for each group, with lower scores indicating greater improvement. *Abbreviations:* MDFI – multidimensional fatigue inventory; IDS-SR – Inventory for Depressive Symptomatology Self Report; sTNFR2 – soluble TNF receptor 2; EBDM – Effort based decision-making.

Using a 2 (treatment group) × 2 (time) × 4 (Effort level) repeated measures ANOVA, we observed a 3-way interaction (F_(3,34)_ = 3.702, *p* = 0.033, η = .098) such that individuals receiving infliximab accepted significantly more effortful options at the higher effort levels (80% and 100%) relative to the placebo group (Fig. 2A). Interestingly, these group differences at the 14-day time-point were driven by both an increase in acceptance of effortful options at 100% effort among patients receiving infliximab (Cohen’s *d* = .30) as well as a decrease among the placebo group (Cohen’s *d* = .33). There were no consistent baseline differences between groups.

To further characterize the impact of infliximab on effort-based choice, we modeled effort discounting by fitting a well-validated computational model of subjective value (see Supplemental Materials for modeling details). Consistent with the effects on effort-based choice behavior, we observed a significant (one-tailed) effect of drug condition on change in free parameter *k* that quantifies the amount that a reward is discounted by the effort required to obtain it (i.e. effort discounting; F_(1,37)_ = 3.950, *p* = 0.028, η = .107) (Fig. 2B). Individuals receiving infliximab showed a significant decrease in *k* parameter values, indicating a reduction in effort discounting.

Across pre-registered self-report measures of anhedonia, we did not observe any significant treatment group × time interactions for symptom improvement (all *p’s* > .1)(see Supplemental Materials). Nevertheless, simple paired t-tests revealed that scores on the IDS-SR anhedonia subscale improved for both the placebo (t_17_ = 2.39, *p* = .029, *d* = .56) and infliximab (t_19_ = 3.93, *p* = .0009, *d* = .88) groups (Fig. 2E), but only the infliximab group showed improvement on the MDFI reduced motivation subscale, (infliximab: t_19_ = 3.55, *p* = .002, *d* = .79; placebo: t_17_ = 1.24, *p* = .232, *d* = .30) (Fig. 2F).

### Associations between change in effort-based decision-making and a marker of TNF activity

In two separate linear regression models, we found that greater decreases in ΔsTNFR2 were associated with larger increases in acceptance rates for 100% effort trials (b=−0.461, *p* = .010) and larger reductions in effort discounting *k* parameter (b = 0.34, *p* = 0.030) (pre-registered as one-tailed) (Fig. 2C–D). Moreover, bootstrapped mediation analysis confirmed that ΔsTNFR2 partially mediated the relationship between drug condition (infliximab or placebo) and Δ100%Effort (95% CI: .007, .249) and Δ*k* parameter (95% CI:−1.15, − .011) (see Supplemental Materials for full mediation results)

### Effects of infliximab on univariate imaging activity during effort-based decision-making and associations with behavioral and immunologic change.

We identified seven contrasts of interest for the EBDM task (see Supplemental Materials). For each ROI, we tested each contrast for effects of drug (infliximab or placebo), and change in circulating sTNFR2 (ΔsTNFR2). We did not observe a main effect of drug in our ROI analyses, but did observe an effect of sTNFR2 on encoding of choice difficulty in dmPFC such that greater engagement of dmPFC during difficult choices was associated with a larger reduction in sTNFR2 (b=−.47, *p* = .013). Moreover, ΔsTNFR2 mediated the association between drug condition and choice-difficulty beta weights (95% CI: .076, 1.94). Full results from other planned contrasts are presented in Supplemental Materials.

**Figure 3:**
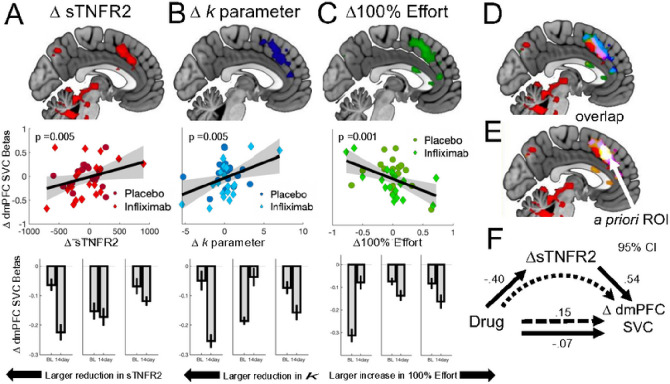
Associations between dmPFC activation and change in inflammatory markers, effort discounting and subjective value encoding at 100% effort. **A.** Larger reductions in sTNFR2 at 14 days were associated with greater reductions in dmPFC subjective value encoding. Top: whole-brain contrast. Middle: shows scatter plot from dmPFC a priori ROI. Bottom: Change in mean dmPFC signal binned by change sTNFR2 **B.** Larger reductions in dmPFC subjective value encoding at 14 days was associated with a reduction in effort-discounting. Top:whole-brain contrast. Middle: shows scatter plot from dmPFC a priori ROI. Bottom: Change in mean dmPFC signal binned by change sTNFR2 **C.**Larger reductions in dmPFC subjective value encoding at 14 days was associated with a greater increase in willingness to accept effortful options at the highest effort level (100%). Top: whole-brain contrast. Middle:shows scatter plot from dmPFC a priori ROI. Bottom: Change in mean dmPFC signal binned by change sTNFR2 **D.** Conjunction of whole-brain maps for DsTNFR2, D*k*, and D100% effort. **E.** Conjunction of whole-brain maps with our *a priori* ROI. **F.** Change in sTNFR2 in dmPFC subjective value encoding mediated the relationship between drug and dmPFC SVC encoding. *Abbreviations:* SVC – SVchosen; sTNFR2 – TNF soluble receptor 2; dmPFC; dorsomedial prefrontal cortex.

We further observed effects of ΔsTNFR2 on dmPFC responses to subjective value of the chosen option SV_chosen_ (see Methods). As with the choice difficulty contrast, a bootstrapped mediation analysis found that ΔsTNFR2 mediated the relationship between drug condition and the change in dmPFC SV_chosen_ encoding (95% CI: − .34, − .03). Additionally, change in SVC encoding mediated the relationship between ΔsTNFR2 and change in effort discounting (Δ*k*) (95% CI: .000; .0048). Finally, using a whole-brain, cluster-corrected analysis, we observed that dmPFC activity was significantly associated with ΔsTNFR2, change in effort discounting (Δ*k*), and change in 100% effort choices (Δ100% Effort). Specifically, change in the dmPFC was more strongly engaged by low subjective value trials in patients with larger sTNFR2 reductions (Fig. 3A), lower effort discounting (Fig. 3B) and more 100% effort choices (Fig. 3C).

All maps showed substantial overlap with each other and with our *a priori* ROI definition for dmPFC (Fig. 3D & 3E). Though not predicted *a priori*, other areas showing whole-brain association included left motor cortex and aspects of medial frontal gyrus (see Supplemental Materials for results).

### Effects of infliximab on multivariate encoding of subjective value.

**Figure 4:**
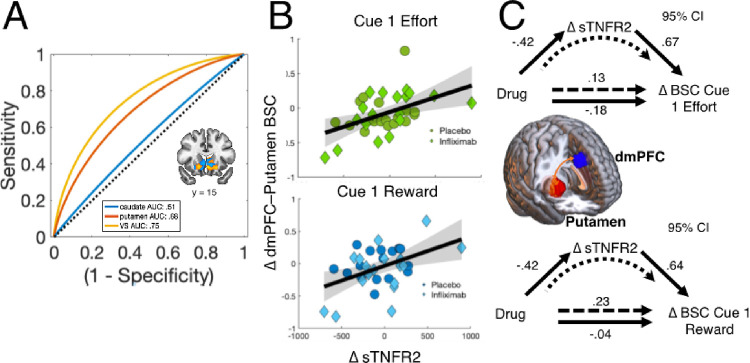
Multivariate classification of drug condition from striatal activity and effects of infliximab on network connectivity. **A.** Receiver operating characteristic (ROC) curves for multivariate classification of drug condition based on regional striatal encoding of predicted subjective value at Cue 1. Estimated pattern of activity in ventral striatum shown in inlay. **B.** Scatter plots showing the association between change in sTNFR2 and change in dmPFC-putamen beta-series correlation (BSC) during Cue1 of the EBDM task when effort information was presented (top) or reward information was presented (bottom). **C.**Schematic diagrams of mediation models tested. Depiction of dmPFC and putamen ROIs shown in inlay. In both cases, change in sTNFR2 significantly mediated the association between drug condition and change in dmPFC-putamen BSC. NB: BSC analyses between dmPFC-ventral striatum and dmPFC-caudate were also tested (not shown). Abbreviations: AUC = area under the curve; BSC = beta-series correlation; VS = ventral striatum; dmPFC = dorsomedial prefrontal cortex.

To further probe for the effects of infliximab on subjective value information, an exploratory multivariate approach was used in three striatal ROIs: ventral striatum, putamen and caudate. Multivariate patterns were estimated using 3-fold cross-validated partial least squares (PLS) regression model on each subject’s difference map for SV_predicted_ value at Cue1 or SV_chosen_ at Cue2. AUC classification between PLS predictions of drug condition and true condition assignment (infliximab and placebo) were then computed (see Supplemental Materials for further details). We observed strong evidence for classification in ventral striatum (AUC = .75, permutation test *p* = 0.007), with moderate classification in putamen (AUC = .68, permutation test *p* = 0.044) and no evidence for classification in the caudate (AUC = .55, permutation test *p* = 0.65) (Fig. 4A). Striatal regions did not show evidence for classification for activity during SV_chosen_. Taken together, these data suggest that changes in the patterns of activity within the ventral striatum and putamen systematically differed between treatment groups, thereby providing evidence of drug-related effects in these areas despite the absence of clear univariate changes.

### Effects of infliximab on corticostriatal network processing

A limitation of the prior analyses is the focus on individual brain areas despite our knowledge that these areas function as an integrated circuit. To examine effects of infliximab on corticostriatal networks, we used an exploratory beta-series correlation (BSC) analysis^51^ to identify how the task-dependent functional connectivity within the network was altered by drug condition and ΔsTNFR2. Given our observed effects on dmPFC at time of choice, this analysis focused on functional connectivity with dmPFC during Cue 1, as this might be the time when partial information was influencing dmPFC connectivity to guide choice behavior. We tested dmPFC beta-series connectivity to ventral striatum (VS), putamen and anterior insula. As above, we found that decreasing sTNFR2 was associated with a significant decrease in beta-series connectivity (BSC) between dmPFC and bilateral putamen (Cue 1 reward: b = .53, *p* = .005; Cue 1 effort: b = .65, *p* = .0002) (Fig. 4B). Additionally, the effect of sTNFR2 mediated the association between drug condition and changing connectivity (Fig. 4C). There was no association with VS, and the change in BSC was significantly stronger for dmPFC-putamen than for dmPFC-VS (Steiger’s Z = 3.46, *p* < 0.001). Taken together, these data suggest that altering sTNFR2 impacted network-level processing of reward and effort information even before full choice information was available.

### Effects of infliximab on “neural signatures” for reward.

**Figure 5:**
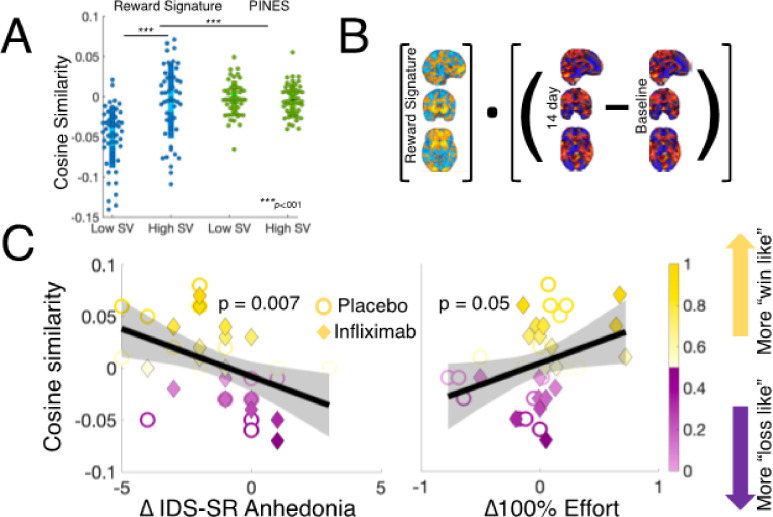
Results of neural signature analyses. **A.** Cosine similarity between the reward signature (blue) and a control signature for picture-induced negative emotions (“PINES”) (green) for low and high subjective value trials during the EBDM task in a sample of 47 healthy controls (see Supplemental Materials for details). Dark shaded area shows SEM and light shaded area shows standard deviation. **B.** Schematic illustration of cosine similarity analysis between change in whole-brain activity during cue 2 of the EBDM task between 14-day and baseline scans and pre-trained “Reward Signature”. **C.**Association of cosine similarity with change in anhedonia (left) and change in 100% Effort choices (right).

In a final set of exploratory analyses, we sought to assess whether a recently validated whole-brain predictive model (aka “neural signature”) that was trained to decode gains versus losses during a monetary reward task^50^ detected treatment-related changes in fMRI signals during EBDM. First, we determined sought to establish that a signature trained to differentiate win and loss feedback would be sensitive to high and low subjective value trials in our task. Using a previously collected sample of healthy controls, we observed a large difference in cosine similarity between high and low subjective value trials for the reward signature (Cohen’s *d* = 1.05, t_46_=−5.024, *p* = 8e-6), with no difference for a control signature developed to classify negatively valanced-stimuli^52^ (Fig. 5A; See Supplemental Materials). Next, we tested whether change in the pattern of whole-brain activity during Cue 2 of the EBDM task was associated with behavioral or symptom change. Consistent with our interpretation of brain-related changes implying greater motivation for reward, we found that Δ100%Effort choices made by participants were associated with a positive shift in cosine similarity with the reward signature such that individuals accepting more 100% effort options at day 14 relative to baseline also exhibited change towards away from a “loss-like” and more towards a “win-like” state (b = .32, p = 0.051). This effect was also associated with a greater reduction in the anhedonia subscale of the IDS-SR (b=−.44, p = .007) (Fig. 5B–C).

## DISCUSSION

Consistent with expectations from our prior theoretical work^21, 53^, we found that relative to placebo, infliximab was associated with an increase willingness to expend effort for rewards. This change was associated with enhanced subjective value encoding for effort-based choices within the dmPFC and the ventral striatum, two key structures that underlie human effort-based decisions^11–13, 54–56^. Of note, however, the observed changes in motivation measured by our behavioral task in infliximab- versus placebo-treated patients were associated with only nominally greater improvement in self-reported motivational anhedonia symptoms in infliximab-treated subjects. Together, these results support the hypothesis that anti-inflammatory strategies may be useful for targeting motivational behavior in depressed patients with evidence of chronic inflammation.

All participants randomized in our study met criteria for current MD and showed evidence of chronic inflammation (CRP > 3.0 mg/L)^57^ and no evidence of infection, autoimmune and inflammatory disorders or other overt causes of inflammation. Substantial prior work has suggested that inflammation may be causally associated with MD^21, 58, 59^, but only for a subset of individuals. This suggests that some MD patients exhibit a vulnerability to chronic, low-grade inflammatory signaling that makes them more likely to develop motivational impairments, which can in turn exacerbate or maintain a depressive episode. The overarching goal of this study was to shed light on the neural circuitry that may underlie this vulnerability.

To that end, the first hypothesis of our study was whether administration of an anti-inflammatory agent like infliximab would lead to increased motivation for rewards. This hypothesis was supported (Figure. 2) with an overall reduction in the discounting of reward by effort, with the strongest effects of infliximab occurring at the highest levels of effort. This suggests that infliximab enhanced individuals’ willingness to exert more strenuous levels of effort for rewards, rather than simply increasing the frequency with which they committed to moderate levels of effort. This effect was driven by changes in both groups; while patients treated with infliximab increased their acceptance (Cohen’s *d* = .30), there was also a general decrease in willingness to expend effort among placebo-treated patients (Cohen’s *d* = − .33); a pattern similar to that observed for placebo in a clinical trial using a monetary reward task and a kappa opioid antagonist^60^.

Regarding neural markers of this behavioral change, neuroimaging analyses highlighted a role for the dmPFC, such that individuals treated with infliximab exhibited stronger univariate responses with increasing choice difficulty and subjective value. The dmPFC has long been known to play a critical role in EBDM, and the observed effects of ΔsTNFR2 showed substantial overlap with our prior work using this fMRI paradigm, demonstrating that dmPFC activity increases as the subjective value of the chosen option becomes less attractive (Fig. 3B).^11, 39^ The neural computations that underlie this region remain a subject of debate^17, 61, 62^. Indeed, some prior work has suggested that the association between dmPFC activity and subjective value may reflect the need to shift strategies^63^ or greater difficulty in adjudicating between the two options^17^, both of which frequently correlate with subjective value. In either case, our results suggest greater engagement in these valuation processes among individuals receiving infliximab. The localization to dmPFC is further notable given prior evidence suggests that inflammatory stimuli directly alter measures of metabolism and activation in this region^24, 27^, and that post-mortem samples from depressed patients show evidence of enhanced immune signaling^64–66^. Taken together, our results highlight dmPFC as a key structure mediating the impact of inflammation on motivated decision-making, and as a potential site of intervention for future treatments targeting the inflammatory subtype.

A possible caveat to this interpretation is that infliximab acted primarily on other brain areas, which led to a behavioral change reflected by changed dmPFC activity. In an effort to address this concern, we included two additional exploratory analyses: multivariate encoding of predictive subjective value (SV_predicted_) at Cue 1 and changes in functional connectivity at Cue 1. The advantage of examining Cue 1 activity is that it allows for interrogation of brain responses to relevant cost/benefit information that are independent of participant choices, which cannot occur until full information is presented at Cue 2. Both these analyses found clear evidence supporting the effect of infliximab and change in TNF signaling on corticostriatal circuit function in a choice-independent manner.

A final exploratory analysis was used to evaluate the effect of infliximab on whole-brain patterns of activity in our task using a previously-validated predictive brain model (aka “neural signature”) that has been shown to discriminate between monetary gains from losses^50^. We found that patients accepting more 100% effortful options and endorsing lower anhedonia exhibited a shift away from a “loss-like” pattern of activity, possibly suggesting that effort costs were perceived to be less aversive.

### Limitations

While the current study had many strengths, including a double-blind, randomized, placebo-controlled design, careful inclusion/exclusion criteria, and state-of-the art neuroimaging methods, several weaknesses warrant comment. First, due to the COVID-19 pandemic and related recruitment challenges, the resulting sample was only powered to detect large and medium effects. A second limitation was use of a single-dose and a relatively brief treatment window, which may have contributed to the lack of a clear treatment group × time interaction for self-reported measures of motivational symptoms. Importantly, the lack of an interaction was largely due to improvement across both groups, rather than an absence of improvement for those receiving infliximab. Moreover, the effect sizes were larger for infliximab for most measures. Given that we observed a clear treatment group × effort discounting interaction, it is possible that patients were beginning to experience more energy and motivation to pursue rewards, but that the placebo effect was still too strong to observe differentiation when patients were asked to report on their symptom experiences.

### Conclusion

A single dose of infliximab resulted in greater willingness to exert effort for rewards, an effect that was reflected in effects on TNF signaling and corticostriatal circuitry, particularly in the dmPFC. These results further support the use of anti-inflammatory treatment strategies for targeting a possible “immune-subtype” of motivational impairments in patients with MD and other psychiatric disorders.

## Figures and Tables

**Table 1:** 

		All Baseline Data	*n*		Infliximab Total Baseline	*n*		Placebo Total Baseline	*n*
**Demographics**									
Age	Mean (SD)	38.97 (8.53)	42		40.02 (9.18)	21		37.91 (7.92)	21
Sex	Female, N (%)	37 (30.10)	42		18 (85.71)	21		19 (90.47)	21
Race									
Asian	N (%)	3 (7.14)	42		0 (0)	21		3 (14.28)	21
Black	N (%)	20 (42.86)	42		8 (38.10)	21		12 (57.14)	21
White	NW	18 (47.62)	42		13 (61.90)	21		5 (23.81)	21
Other	N (%)	0 (0)	42		0 (0)	21		0 (0)	21
BMI	Mean (SD)	33.46 (6.08)	36		33.48 (5.62)	18		33.44 (6.68)	18
									
**Clinical Characteristics**									
Bipolarity Index	mean (SD)	25.68 (13.06)	37		29.39 (15.71)	18		22.05 (9.04)	19
Bipolarity Index >= 50	N (%)	2 (5.41)	37		2 (11.11)	18		0 (0)	19
Co-Morbid Anxiety	N (%)	24 (58.54)	41		13 (61.90)	21		11 (55.00)	20
Co-Morbid PTSD	N (%)	4 (9.76)	41		1 (4.76)	21		3 (15.00)	20
									
hs CRP (mg/L)	Median	3.62	42		3	21		4.11	21
									
IDSSR	Mean (SD)	35.60 (9.79)	42		36.67 (10.84)	21		34.52 (8.75)	21
IDSSR Anhedonia	Mean (SD)	4.59 (1.93)	42		4.67 (2.03)	21		4.52 (1.86)	21
MDFI RM	Moan (SD)	14.76 (3.23)	41		15.43 (3.26)	21		14.02 (3.12)	20
MDFI RA	Mean (SD)	13.56 (4.03)	41		13.67 (3.53)	21		13.45 (4.59)	20
									
**EBDM Performance**									
% Total Chocies	Mean (SD)	72.30 (13.68)	42		73.36 (14.33)	21		71.24 (13.26)	21
*K* parameter	Mean (SD)	2.09 (1.63)	42		2.13 (1.76)	21		2.06 (1.55)	21
Model fit (BIC)	Mean (SD)	27.34 (12.94)	42		24.77 (12.79)	21		29.92 (12.88)	21
% Time Outs	Mean (SD)	2.22 (3.54)	42		2.44 (3.26)	21		2.00 (3.87)	21
% Post Effort Switches	Mean (SD)	8.87 (11.86)	40		5.92 (8.63)	20		11.82 (13.99)	20
									
